# Effects of the Pregnancy and Newborn Diagnostic Assessment (PANDA) App on Antenatal Care Quality in Burkina Faso: Protocol for a Cluster Randomized Controlled Trial

**DOI:** 10.2196/37136

**Published:** 2023-08-09

**Authors:** Abou Coulibaly, Séni Kouanda

**Affiliations:** 1 Département Biomédical et Santé Publique Institut de Recherche en Sciences de la Santé Ouagadougou Burkina Faso; 2 Institut Africain de Santé Publique Ouagadougou Burkina Faso

**Keywords:** telemedicine, PANDA, pregnancy and newborn diagnostic assessment, quality, antenatal care, Burkina Faso, trial, pregnancy, pregnant, newborn, diagnostic, mobile app, prenatal care, randomized trial, first trimester, postpartum, qualitative research, maternity, prenatal, antenatal, mobile phone

## Abstract

**Background:**

The Pregnancy and Newborn Diagnostic Assessment (PANDA) system is a digital clinical decision support tool that can facilitate diagnosis and decision-making by health care personnel in antenatal care (ANC). Studies conducted in Madagascar and Burkina Faso showed that PANDA is a feasible system acceptable to various stakeholders.

**Objective:**

This study primarily aims to evaluate the effects of the PANDA system on ANC quality at rural health facilities in Burkina Faso. The secondary objectives of this study are to test the effects of the PANDA system on women’s satisfaction, women’s knowledge on birth preparedness and complication readiness, maternal and child health service use, men’s involvement in maternal health service utilization, and women’s contraception use at 6 weeks postpartum. Further, we will identify the factors that hinder or promote such an app and contribute to cost-effectiveness analysis.

**Methods:**

This is a randomized controlled trial implementing the PANDA system in 2 groups of health facilities (intervention and comparison groups) randomized using a matched-pair method. We included pregnant women who were <20 weeks pregnant during their first antenatal consultation in health facilities, and we followed up with them until their sixth week postpartum. Thirteen health centers were included, and 423 and 272 women were enrolled in the intervention and comparison groups, respectively. The primary outcome is a binary variable derived from the quality score, coded 1 (yes) for women with at least 75% of the total score and 0 if not. Data were collected electronically using tablets by directly interviewing the women and by extracting data from ANC registers, delivery registers, ANC cards, and health care records. The study procedures were standardized across all sites. We will compare unadjusted and adjusted primary outcome results (ANC quality scores) between the 2 study arms. We added a qualitative evaluation of the implementation of the PANDA system to identify barriers and catalysts. We also included an economic evaluation to determine whether the PANDA strategy is more cost-effective than the usual ANC strategy.

**Results:**

The enrollment ran from July 2020 to January 2021 due to the COVID-19 pandemic. Data collection ended in September 2022. Data analyses started in January 2023, ended in June 2023, and the results are expected to be published in February 2024.

**Conclusions:**

The PANDA system is one of the most comprehensive apps for ANC because it has many features. However, the use of computerized systems for ANC is limited. Therefore, our trial will be beneficial for evaluating the intrinsic capacity of the PANDA system to improve the quality of care. By including qualitative research and economic evaluation, our findings will be significant because electronic consultation registries are expected to be used for maternal health care in the future in Burkina Faso.

**Trial Registration:**

Pan-African Clinical Trials Registry (PACTR) PACTR202009861550402; https://pactr.samrc.ac.za/TrialDisplay.aspx?TrialID=12374

**International Registered Report Identifier (IRRID):**

DERR1-10.2196/37136

## Introduction

### Background

Antenatal care (ANC) is one of the strategies that contributes to reducing maternal and neonatal mortality. In the early 2000s, the concept of ANC was introduced and refocused to be more efficacious [1-3]. Essential activities such as the prevention of anemia and malaria in pregnant women, preparation for delivery and obstetric emergencies, and advice on good nutrition for women and newborns have found their place in the new vision of ANC [4]. In addition, the World Health Organization (WHO) guidelines recommend 8 antenatal visits to the clinic so that each point of contact will ensure a smooth and safe pregnancy [5]. According to the WHO, increasing the frequency of ANC visits for women and adolescents is associated with a reduced risk of stillbirths. These visits provide additional opportunities to detect and manage potential problems [5]. Additionally, regular antenatal visits to the clinic increase the chance of delivering at a health facility [6].

Despite the importance of ANC, data on the use of services by pregnant women remain scarce in sub-Saharan Africa [7-9]. Several reasons could explain this situation, including the quality of care and satisfaction of pregnant women with health services. Nikiema et al [10] showed that the different steps of ANC are not carefully adhered to, ignored, or misunderstood by health care providers in Burkina Faso. Tiembré et al [11] who evaluated the quality of ANC in Grand-Bassam (Ivory Coast) reported that the interpersonal communication skills of the health care providers with patients were poor. It is essential to systematically follow the different stages of ANC to provide quality care to pregnant women. One solution to improving the quality of ANC is the standardization of care, obliging health workers to systematically follow all the steps of ANC consultation [12-14]. Therefore, the use of mobile apps and telemedicine seems appropriate. The WHO insists on using mobile apps to provide maternal and child health care because these apps play an essential role in improving the quality of health care services [15]. In 2013, the World Health Assembly passed numerous resolutions on information and communication technologies in the health care sector. The use of apps in health care has been noted to be related to reduced health care costs, improved quality, and equitable access to health services [15]. The application of information and communication technologies in the health sector includes client education and behavior change, medical record management, electronic decision support, quality care in health services, human resource management, and supply chain management [16].

Concerning decision support and quality of care, ANC has been a particular focus of mobile app interventions. However, different studies [12-17] show contrasting results. In 2018, Chen et al [18] conducted a systematic review of 245 studies, including 51 randomized controlled trials, on the effectiveness and appropriateness of mobile apps for maternal and child health care between 2011 and 2016. Their results showed that mobile apps had no effect on the quality of health care in approximately half (43%) of the randomized controlled trials.

The Pregnancy and Newborn Diagnostic Assessment (PANDA) system [19,20] is a digital clinical decision support tool that facilitates diagnosis and decision-making by health care personnel in ANC. Feasibility and acceptability studies of the PANDA system have been conducted in Madagascar and Burkina Faso [14,21]. The study [21] conducted in the Koupéla district of Burkina Faso in 2016 with the nongovernmental organization, Enfants du Monde, Switzerland, showed that the PANDA system is a feasible tool for improving the quality of care for pregnant women and for collecting detailed information in the electronic form. The women in that study [21] expressed high satisfaction with the antenatal visits using this telemedicine system except for the average duration of ANC (women found that the average duration of ANC is high with the PANDA app), which would increase with the app use. The health care workers expressed their satisfaction with the service improvements and noted the different steps of ANC according to the recommendations of Burkina Faso. In the PANDA app, the different steps of ANC are presented in an imposed chronology to the health care provider. The completeness of the data for all fields conditions the passage from one stage to the next. Therefore, it is impossible to omit information. Following these encouraging results, the Ministry of Health of Burkina Faso expressed interest in using the PANDA telemedicine system for maternal and neonatal health care to improve the quality of care. Hence, we plan to implement a randomized controlled trial to evaluate the effectiveness of PANDA in improving the quality of ANC.

### Study Objectives and Hypothesis

The objective of this study was to evaluate the effects of the PANDA system on the quality of ANC in Burkina Faso. We hypothesize that the PANDA telemedicine system improves (1) the quality of ANC provided to pregnant women, (2) women’s satisfaction, (3) women’s knowledge on birth preparedness and complication readiness, (4) maternal and child health service use, (5) men’s involvement in maternal health service utilization, and therefore (6) postpartum contraception use.

### Study Setting

This study was conducted in Burkina Faso, where the ANC rate varies according to the area of residence. The 2010 Demographic and Health Survey and Multiple Indicator Cluster Survey (Burkina Faso Enquête Démographique et de Santé et à Indicateurs Multiples [EDSBF-MICS IV]) showed that 1.5% and 5.7% of the pregnant women did not attend antenatal visits in the urban and rural areas, respectively [22]. Moreover, according to the EDSBF-MICS IV, only 33.7% of the pregnant women have received at least 4 ANC consultations as recommended by the WHO. Only 41.2% of the women had their first ANC visit in the first trimester of pregnancy, and the median number of months of pregnancy at the first ANC visit was 4.4 months at the national level [22]. Among the women who received ANC for the most recent birth 5 years before 2010, only slightly more than half (53%) had been informed about the signs of pregnancy complications. In ANC, it is crucial for pregnant women to know the pregnancy danger signs. The EDSBF-MICS IV also showed that almost all women (95%) received ANC from a trained provider, and the majority (61%) of these trained providers were auxiliary birth attendants. Midwives provided ANC for 17% of the women, and nurses examined almost the same proportion of women (16%). Approximately one-third of the women (34%) had at least 4 antenatal visits, which aligns with the Burkina Faso Ministry of Health’s recommendations. There were significant differences between the areas of residence in Burkina Faso: in Ouagadougou (Burkina Faso’s capital), 54% of the women attended the 4 recommended visits compared with 38% in other cities and only 31% in rural areas [22]. The consequences of this low access to maternal, newborn, and child health services are high maternal and infant mortality rates. One in 8 children die before 5 years, and the maternal mortality ratio was 341 deaths per 100,000 live births in 2010 [22]. We chose the health district of Koupéla (Centre-East Region of Burkina Faso) because of its proximity to Ouagadougou. At that time, there were no ongoing ANC interventions in this district. The other reason for choosing this district is that the PANDA feasibility test was conducted in this district in 2016.

## Methods

### Conceptual Framework of This Study

Our research project consists of implementing an intervention called the PANDA telemedicine system. This study phase, which we call the interventional phase (as it follows the design and feasibility phases of the intervention), involves 2 groups of health facilities in the same district (an intervention group and a comparison group). We compared the effects of the intervention with those of the usual ANC offered in the comparison group through a cluster randomized trial. The interventional phase also employs qualitative research and a cost-effectiveness study at the end of the trial. The qualitative research identifies the barriers and catalysts of using the PANDA system for ANC in the intervention group. The cost-effectiveness analysis provides information on the costs of implementing the PANDA system in Burkina Faso. The expected effects of the app are improvement in (1) the quality of ANC provided to pregnant women, (2) women’s satisfaction, (3) women’s knowledge of birth preparedness and complication readiness, (4) use of maternal and child health services, (5) men’s involvement in maternal health service utilization, and (6) contraception use at 6 weeks postpartum. The long-term impact of this app is the reduction of maternal and neonatal mortality.

### Cluster Randomized Controlled Trial

#### Study Design

**Figure 1:**
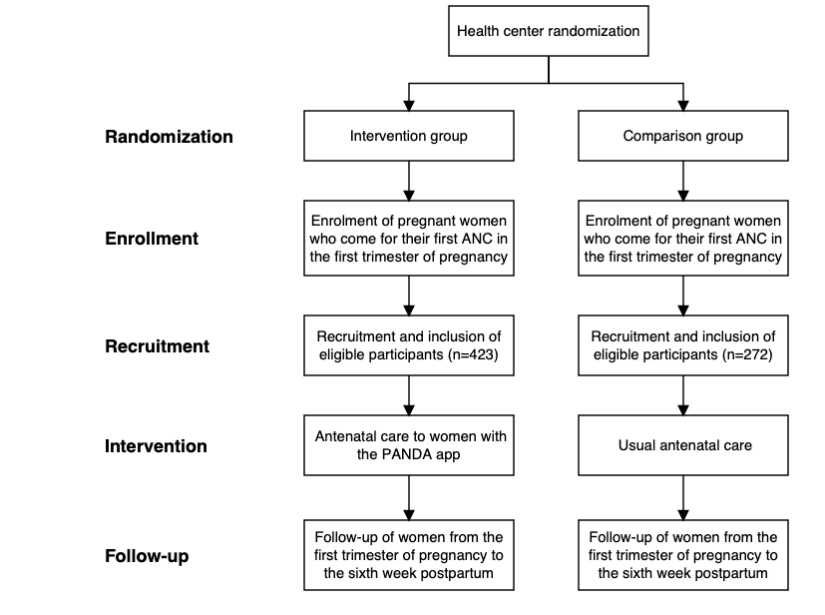
Study design. ANC: antenatal care; PANDA: Pregnancy and Newborn Diagnostic Assessment.

In this study, the intervention is the PANDA telemedicine system. It involves 2 groups of health facilities in the same district (an intervention group and a comparison group). The intervention group offered ANC using the PANDA app installed on tablets. Each health facility in this group had at least one tablet. The health centers in the comparison group offered ANC as usual (the PANDA app was not used in these health facilities). The rationale for implementing a cluster randomized trial is that the intervention (training and use of the PANDA system) cannot be implemented at the individual (pregnant woman) level but only at the health center level. The study design is shown in Figure 1.

#### Participant Eligibility Criteria

The participants were pregnant women with gestational age between 14 and 20 weeks of amenorrhea, regardless of age, residence, and socioeconomic level. They were screened for eligibility for this study: first by health care workers on arrival and second by the data collectors of this study. Any pregnant woman who met the following criteria was eligible for this study: (1) <20 weeks of gestation, (2) belonging to the health facility area, (3) no plan to travel in the next 9 months (from inclusion in this study to 6 weeks postpartum), (4) not taking part in any other ongoing research, (5) volunteering to participate, (6) being in a clinical and obstetrical condition that allows delivery at the health center (no need to refer to the next level), and (7) has the intention to deliver in that health center.

#### Interventions

**Figure 2:**
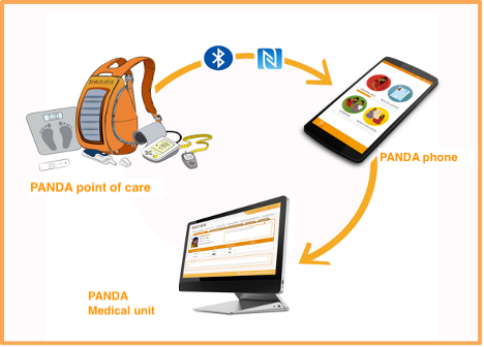
The Pregnancy and Newborn Diagnostic Assessment system. PANDA: Pregnancy and Newborn Diagnostic Assessment.

PANDA is a telemedicine system developed through a private-public partnership in Italy, with funding from the European Union, in collaboration with the WHO. The PANDA system was designed to meet the health care needs of women during pregnancy in resource-scarce settings by expanding and standardizing ANC according to the WHO guidelines. The PANDA system, designed for use by low-skilled health care workers such as community health care workers, supported remotely by skilled personnel, aims to standardize and improve the quality of ANC visits. In addition, the system can automatically generate and provide the data necessary to measure its performance. The original PANDA system, as designed for use by community health care workers, consists of 3 integrated components (see Figure 2).

1. The PANDA phone: This smartphone with an Android app allows for a standardized and quality antenatal visit by using pictograms according to the national and WHO guidelines. The health care workers enter the women’s data and obstetrical and medical-surgical history into the system. The system also includes pages for medical tests to ensure that health care workers perform these tests. At the end of the visit, the PANDA phone sends the data and test results to the medical unit.
2. The PANDA point of care: A series of medical tests are performed to screen for anemia, syphilis, HIV, malaria, eclampsia, diabetes, and malnutrition. The test results are entered manually or via Bluetooth into the PANDA phone.
3. PANDA medical unit: A database system receives data from the PANDA phone, establishes diagnoses and the management of pregnant women, and maps pregnancies in the region. The PANDA medical unit in the maternity ward typically consults this database.

The PANDA system used in our proposed study is like the system used in the pilot feasibility and acceptability study in 2016 [21]. It has already been adapted to the Burkinabè context. The PANDA system will be integrated into the Burkinabè health system and used by the Ministry of Health’s care providers in the health center under the supervision of the district management team. The PANDA app and the PANDA medical unit will be readapted to the new national norms and standards for ANC, which are currently under revision based on the new “WHO Recommendations on Antenatal Care for a Positive Pregnancy Experience” [4]. To adapt the tool to other telemedicine systems in practice in Burkina Faso, tablets have replaced smartphones. A user’s manual for the PANDA app was made available to the stakeholders for the judicious use of the app. We organized several forms of training to explain better the use of the PANDA app (user training at the health center and medical unit levels).

#### Outcomes

The primary outcome is a binary variable derived from the quality score. It will be coded 1 (yes) for women with at least 75% of the total score and 0 if not (Table 1). The quality score considers all the components of good ANC according to the gestational age of the pregnancy. The different components are grouped into 7 main dimensions with items. These items are derived from Burkina’s guidelines on ANC [23]. The categorization is that each woman has a total score for each ANC (from the first consultation to the last).

**Table 1:** Summary of the components of quality antenatal care.

Item (1 if yes, 0 if no)	Data source
Antenatal consultation performed	Woman’s health card reports
Partner’s presence at the consultation	Item asked to the woman
Possession of a verified health record	Woman’s health card reports
Pregnancy test performed	Woman’s health card reports
Date of last period requested	Item asked to the woman
Probable date of delivery given to the pregnant woman	Item asked to the woman
Hypertension was investigated	Item asked to the woman
Asthma was investigated	Item asked to the woman
Testing for sickle cell disease	Item asked to the woman
Diabetes was investigated	Item asked to the woman
Surgical history was obtained	Item asked to the woman
Number of pregnancies was queried	Woman’s health card reports
Number of deliveries was queried	Woman’s health card reports
Number of abortions or miscarriages was queried	Woman’s health card reports
Number of caesarean deliveries was queried	Woman’s health card reports
Number of living children was queried	Woman’s health card reports
Alcohol consumption queried	Woman’s health card reports
Tobacco use queried	Woman’s health card reports
Sugar and albumin levels in the urine measured	Woman’s health card reports
Syphilis test performed	Woman’s health card reports
An HIV test was offered to the woman	Woman’s health card reports
HIV test was performed	Woman’s health card reports
Blood pressure measurement	Woman’s health card reports
Fetal heart sounds recorded	Woman’s health card reports
Search for edema of the feet and face	Woman’s health card reports
Weight gain	Woman’s health card reports
Temperature checks	Item asked to the woman
Physical examination: uterine height measured	Woman’s health card reports
Physical examination: vaginal touch performed	Woman’s health card reports
Physical examination: decision on obstetrical prognosis (normal pelvis, borderline pelvis, etc)	Woman’s health card reports
Tetanus vaccination verified and performed	Woman’s health card reports
Hepatitis B vaccination verified and performed	Woman’s health card reports
Antianemia prophylaxis (iron–folic acid) prescribed	Woman’s health card reports
Antianemia prophylaxis: woman explains dosage correctly	Item asked to the woman
Prescribed antimalarial prophylaxis	Woman’s health card reports
Malarial prophylaxis: woman explains dosage correctly	Item asked to the woman
Danger signs of pregnancy explained to the woman	Item asked to the woman
Place of delivery discussed	Item asked to the woman
Importance of exclusive breastfeeding explained	Item asked to the woman
Early breastfeeding of the baby has been advised	Item asked to the woman
Maternal nutrition: good feeding practices discussed	Item asked to the woman
Postpartum contraception was discussed	Item asked to the woman
Use of an impregnated net was explained	Item asked to the woman
Newborn care was explained	Item asked to the woman
The childbirth preparation plan was discussed	Item asked to the woman
The woman was asked to raise concerns where appropriate	Item asked to the woman
The next meeting was negotiated	Item asked to the woman
Summary of the antenatal care written in the woman’s health record	Woman’s health card reports

The secondary outcomes to measure include the satisfaction of pregnant women (measured at each ANC), women’s knowledge on birth preparedness and complication readiness (measured in the first trimester and just before delivery), use of maternal health services, men’s involvement in health service use (defined as the proportion of men who accompany their wives to the health centers for ANC sessions and delivery and the proportion of men who attended an ANC session or delivery), and finally, the women’s use of postpartum family planning (measured at the sixth week postpartum).

#### Other Variables

We collected women’s sociodemographic characteristics such as women’s age, education, marital status, residence, and occupation. We also collected data on women’s autonomy (Multimedia Appendix 1). We asked the women, in some situations, regarding who makes the final decision: herself, her husband, mother-in-law, father-in-law, or any other person. She could name more than one person if the decision was made collectively. These autonomous situations or items could be small household expenses, major household expenses, women’s expenses, women’s job, buying children’s clothes, where to go for advice if her child is ill, where to go to see a doctor if she is ill, buying medicines, visiting her parents (when or where), number of children in her household, and using the family planning method. The woman’s autonomy variable will be derived from these variables (composite index) and will have 3 modalities (low autonomy, moderate autonomy, and high autonomy).

#### Sample Size

In addition to the desired statistical power and percentage of potential lost to follow-up, the calculation of the required size considered several statistical features inherent to the design of the study, such as the number of participants per cluster, the intraclass correlation coefficient (ICC), and the inflation factor [24-27]:

1. Number of participants: To be practical and comfortable with data collection, we considered 40 women per health center, excluding those lost to follow-up.
2. ICC: This measures the extent of the cluster effect and assesses the degree of similarity between the individuals within a cluster. It is based on the relationship between the intercluster variance and the intracluster variance. For this reason, the sample sizes must be increased to account for the clustering effect. In particular, the value of ICC depends on the unit of randomization and the type of outcome. In our case, we have women likely to come from the rural areas of several health zones. Given the composition of health care workers in rural health centers (midwives and birth attendants), it is reasonable to assume that there will be slight variations between women moving from one health center to another. For this, we propose an ICC of 0.015.
3. Inflation factor: The increase required to account for the cluster effect is influenced by ICC and cluster size. The standard sample size was increased by an inflation factor equal to [1 + (n – 1) ρ], where n is the average cluster size and ρ is the estimated ICC. By numerical application, inflation factor = 1.585.
4. The desired statistical power was 80%.
5. The margin of error was 5%.
6. The estimated proportion of those lost to follow-up was set to 10%.

Using Stata 15.1 software [28], health centers needed to detect a difference of at least 15% (80% in the intervention group vs 65% in the control group) in the proportion of women with an excellent ANC quality score in 6 health centers in each group. Considering the loss to follow-up proportion at 10%, the minimum final size required is 264 women in each group with 12 health centers, except the urban health facility. The hypothetical proportion of 65% in the comparison group was derived from a study conducted in Ghana in 2019 by Baafi et al [29].

#### Participant Recruitment

The health care providers recruited the participants during the ANC sessions after history taking and physical examination. After screening a potentially eligible woman, her consent (free and informed decision) to participate in this study was obtained after she received all the necessary information from the data collector.

#### Randomization

Facilities were randomized using a matched-pair method. Health centers were selected by identifying 6 pairs of comparable health centers. The matching criteria were (1) number of total ANC visits in the year before the trial, (2) number of total deliveries in the year before the trial, (3) number of total postnatal care visits in the year before the trial, (4) number of women of childbearing age in the health center area, (5) total number of health care workers in the health center as well as the distribution by profile (midwives, nurses, and auxiliary birth attendants), and (6) size of the population served. These matching variables allow us to obtain facilities within each pair that are similar to each other regarding these matching factors. Within each pair, the center that received the PANDA app was chosen randomly using the draw of 1 health center from each pair; the names of the health centers were previously arranged in individual envelopes and sealed. The drawer was different from the one who prepared the envelopes and did not attend the sealing. The draw, therefore, was carried out 6 times. All consecutive and eligible participants were included in the clusters.

To assess women in the semiurban environment (city of Koupéla), we included the health center in Koupéla, which unfortunately could not be matched to another city, given its size and characteristics. To minimize the likelihood of cross-group contamination, facilities are chosen to ensure they are far enough apart. Moreover, women generally did not move from one center to another due to the distance between the health centers. They usually visit the same health center for ANC during their pregnancy. We included only the women who were sure they would realize all their ANC visits in the health center where they were included in this study. Lastly, no staff move was noted during the study between the 2 arms. The health centers selected as the study sites are shown in Table 2.

**Table 2:** Study sites.

	Health center
Pair 1	Baskouré and Kalwêga
Pair 2	Boangtenga and Zaogo
Pair 3	Dialgaye and Yargo
Pair 4	Baadtenga and Sampongho
Pair 5	Gounghin and Ligdi-Malguem
Pair 6	Kabéga and Zéguédéga
One semiurban center	Koupéla health center

#### Implementation

After the health care workers were trained in the intervention group’s health centers, supervisions were organized throughout the data collection period to verify the implementation of the intervention.

#### Blinding

Given our intervention’s nature, blinding the participants and the research team was impossible.

### Data Collection Methods

Data collectors were trained before the inclusion of participants on all the procedures of inclusion, follow-up, and how data must be collected (data sources). They were not health care workers. Therefore, the study procedures were standardized across all sites. Data were collected from women on the day of inclusion and at each successive ANC, up to the sixth week after delivery. One data collector was assigned to 2 neighboring health centers for the duration of the study (1 intervention group health center and 1 comparison group health center). The questionnaires were administered to the participants immediately after the antenatal visit, after the delivery, and after each postpartum visit. The interviewer visited the health center according to the ANC schedule. A field coordinator was recruited and responsible for checking all the data collection steps, including the recruitment of participants.

The participant’s knowledge was measured at the study inclusion and at the end of pregnancy. This knowledge covered topics such as the danger signs of pregnancy, signs of labor starting, danger signs after childbirth for the woman, and newborn’s danger signs. All these signs are normally discussed with the woman at each ANC visit to improve her knowledge to stimulate early consultation if any one of the following signs occur:

1. Danger signs of pregnancy: vaginal bleeding, fever, intense intractable headache, difficulty breathing, convulsions and loss of consciousness, intense abdominal pain, non–bloody vaginal discharge, lack of fetal movement, and feet and hands edema.
2. Signs of the onset of labor: loss of water, painful and regular contractions, and discharge of red and viscous mucus.
3. Newborn danger signs: fever, hypothermia, convulsions, inability to breastfeed, vomiting, pus or bleeding from the cord, purulent discharge from the eyes, jaundice, hypotonia, unexplained crying, abdominal pain, bloating, diarrhea, and difficulty breathing.
4. Danger signs after childbirth for the woman: convulsions, heavy vaginal bleeding, intense abdominal pain, intense headaches, dizziness, rapid or difficult breathing, fever, bad odor discharge from the vulva, and paleness of the mucous membranes.

**Figure 3:**
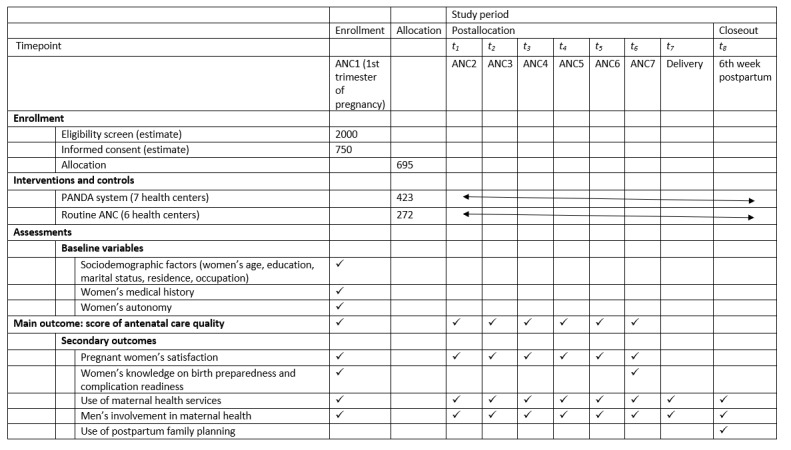
Schedule of participants' enrollment, allocation, interventions, and assessments in the randomized controlled trial. ANC: antenatal care; PANDA: Pregnancy and Newborn Diagnostic Assessment.

For each group of signs, there was a proportion of women who knew no signs and a proportion of women who knew at least 3 signs. Furthermore, we measured women’s birth preparedness by asking the woman if she had identified a health facility for her delivery or in case of emergency, if she had identified a companion of choice for labor and childbirth, or in case of emergency, if she had planned the financing of the main expenses related to her delivery and if she had identified one means of transport to reach the health facility for the delivery. Those who took precautions for each of the 4 questions scored 1 for each question and 0 if the woman took no precautions. The use of maternal and child health services was measured by the number of ANC visits attended by the woman, place of delivery, postnatal consultation on days 6-10, and postnatal consultation in 6-8 weeks. The different variables with their measurement periods are presented in Figure 3.

For this study, data were collected using a closed questionnaire by directly interviewing the women and extracting data from ANC registers, delivery registers, ANC cards, and health care records. To understand the satisfaction of pregnant women, we use a satisfaction scale consisting of very satisfied, satisfied, neutral, not very satisfied, and not at all satisfied, which will be rated as 4, 3, 2, 1, and 0, respectively. The data collector explained to the woman that she is free to give the number of green stars corresponding to her satisfaction level for each subitem listed in Textbox 1 below. The image is shown and explained to the woman in order to rate her satisfaction. The number of green stars chosen correspond to her level of satisfaction with that subitem. The maximum score for each subitem is 4 and the minimum is 0. This satisfaction was measured at each antenatal visit for each woman enrolled in the research. It should be recalled that the data collectors were women without health care training who were recruited and assigned to each health center. Data collectors did not interfere in the care relationship with the women.

**Items and subitems for measuring the satisfaction of pregnant women.**

**Women’s satisfaction with interpersonal relations with staff (5 subitems)**

- Welcoming
- Respecting the confidentiality of consultation with the health care provider
- Respecting privacy during consultation
- Respect and courtesy of the health care providers during antenatal care
- Goodbye from staff

**Quality of service (9 subitems)**

- Waiting time
- Overall time spent in the health center
- Other times to wait for test results
- Health care provider skills
- Explanation of iron–folic acid dosing
- Explanation of malarial prophylaxis
- Explanation of preventive measures against malaria
- Explanation of women’s diet
- Explanation of different complementary examinations

### Data Management

Data collection was performed electronically by using tablets. Only the identifying information of the participants was collected on paper to maintain the anonymity of the women in the different databases. In both groups, registers, health records, ANC cards, and women’s self-report were used for data collection. We developed a data quality assurance plan for the duration of the trial. Implementation was under the supervision of the research team. Confidentiality of the data was ensured by protecting devices with passwords kept in a secure location. A data entry program was developed using the CSEntry Android app of Census and Survey Processing System software [30]. The data manager exported the data to Stata 15.1 for correction and use. We implemented the following measures to ensure effective data management:

1. The information collected during the various contacts with women was verified several times at different levels. Data collectors were responsible for ensuring that all data collected were accurate. They reviewed all the completed forms before uploading them to the server to guarantee completeness and resolve inconsistencies.
2. All data were stored on a server compatible with good clinical practice, and data transmission will be encrypted to ensure data integrity and patient confidentiality. The access to data management was password protected, and only authorized users had access.

### Statistical Methods

Quantitative data were extracted and transferred to Stata 15.1 for analysis. We will perform an intention-to-treat and per-protocol analysis of the cluster randomized trial. The unit of analysis will be women. The analysis will compare the intervention and comparison groups’ unadjusted and adjusted primary outcomes results. Unbalanced factors between the 2 study groups will be used to adjust the intervention effects on the study outcomes. These factors will be women’s sociodemographic factors and women’s autonomy. The means and proportion comparison tests for independent samples will be used at the 5% threshold. In case of nonnormality of a quantitative variable (satisfaction scores, knowledge scores, etc), robust or Poisson regression will be used.

### Data Monitoring

Since the participants were not given an experimental drug, we did not conduct an interim analysis to determine whether to stop. The team was supervised every month. The management focused on aspects such as the quality of the collected data, careful adherence to every step of the protocol and validated methods, and using the PANDA app. In addition, monthly meetings were held online with all technical and financial partners to resolve difficulties. The supervision and meeting reports were produced and archived systematically.

### Ethics Approval

This trial was retrospectively registered with the Pan-African Clinical Trials Registry under PACTR202009861550402 on September 21, 2020. The Burkina Faso Health Research ethics committee approved the research protocol for this trial under approval 2020-3-051. This study does not introduce any new drug treatments. The PANDA system was designed by the standards and protocols in force in Burkina Faso. Any problems identified will be managed according to the procedures and practices in the health system. Adult participants (at least 18 years old) were included after obtaining their informed consent. Minor participants were included by obtaining their assent and parental consent. In the case of minor participants, informed consent of the parent or the respective legal guardian was obtained in conjunction with informed permission of the underage participant (younger than 18 years). The interviewers assigned to the various health centers were responsible for obtaining consent. This process was carefully checked during the field supervision of the research team. All questionnaires in the database did not contain any information that could be used to identify a woman formally. The participants’ phone numbers and identities were recorded in a physical document, allowing the interviewer to locate the woman, if necessary, to administer a questionnaire.

### Qualitative Analysis of Factors Hindering or Facilitating Implementation

We conducted a qualitative evaluation of the implementation of the PANDA system to identify the interactions (positive or negative) between the intervention and the implementation environment. The target population comprised the intervention group and the resource personnel. The intervention group included women who had recently given birth, providers of the health centers where the PANDA system was used, and resource persons. With women, we measured mainly their perceptions of the quality of care and interactions with health providers. The health care providers were interviewed to give their opinion about the PANDA system’s technical aspects, interactions with women, and perceptions of the quality of the medical procedures provided. Given their positions in this research, the resource persons were the key informants. They were the district management team, managers of the medical unit, Directorate of Health Information Systems, Directorate of Family Health, and members of 2 nongovernmental organizations, namely, Enfants du Monde, Switzerland, and the Private and Community Initiative for Health and the Response to HIV/AIDS in Burkina Faso.

Following the reasoned approach, we performed 4 focus groups with women who had recently given birth (1 focus group per center in 4 health centers), 22 in-depth individual interviews, and again with women who had recently given birth in the 7 health centers (3 interviews per center); 18 in-depth individual interviews were conducted with the health workers of the 7 health centers (2 per center, including the medical unit). However, given the size of the CSPS Urbain (the only semiurban center), the number of health workers interviewed was 4. We also conducted 4 in-depth individual interviews with the technical and financial partners. In total, 44 individual interviews and 4 focus groups were performed.

The qualitative component used the semistructured interview [31]. The women were recruited in a reasoned manner from among those included in the study—mainly the last women enrolled in this study to give birth to guarantee the freshness of memory. In addition to the primary data, secondary data were collected through activity reports (training missions since the beginning, supervision missions conducted by the research team, etc), budgets allocated to program activities, national health policy documents, PANDA app user guide, etc. The explored themes were as follows: the satisfaction of the participants in the intervention group, the acceptability of the intervention to health care workers, and the barriers and catalysts for an efficient and effective implementation of the PANDA system.

Content analysis [32] was used as the data processing and analysis technique. The digitally collected data were transcribed into French and entered into Microsoft Word. The transcribed data were analyzed thematically using a manual approach with the help of analysis tables. The analysis model used was the strengths-weaknesses-opportunities-threats model. It enabled us to identify the interactions (positive or negative) between the intervention on the one hand and the implementation environment on the other. These diversions will allow us to consider the necessary readjustments for greater efficiency and effectiveness of the intervention.

### Economic Evaluation

An economic evaluation was added to this research to determine whether the PANDA strategy is more cost-effective than the usual ANC strategy in Burkina Faso’s context. The economic assessment collected data from pregnant women (expenses related to antenatal visits), health agents, and financial partners (costs of designing the app, training costs, acquiring equipment, etc). The direct prices included drugs and consumables for health centers. For women, the costs included direct costs such as transport costs for the health center visit and indirect costs, especially the monetary value of the time spent visiting the antenatal service. As ANC is currently free for women, no expenses of ANC-related care are paid by women in principle.

The economic evaluation data were analyzed using Stata software (version 17.1; StataCorp LLC) for the descriptive analyses and R software (version 4.0.2; R Foundation for Statistical Computing) for the Monte Carlo simulations. We also adapted the WHO CostIt tool [33] to calculate the costs according to the ingredients approach [34] for each cost category. Incremental cost-effectiveness was examined based on comparisons of costs and effects between the study groups. We conducted a probabilistic sensitivity analysis by testing the effect of simultaneous variations in several parameters by using Monte Carlo simulations to generate plausible values from the baseline parameter distributions. A total of 10,000 iterations were generated by R programming. A cost-effectiveness acceptability curve was developed based on the willingness to pay to gain a percentage of ANC quality and compared to the gross domestic product per capita of Burkina Faso in 2020.

## Results

Participant recruitment in this study was influenced by the COVID-19 pandemic. Although recruitment was planned to start in May 2020, the inclusion finally began only on July 12, 2020, and was done progressively in different health centers included in this study. Participant selection continued until the end of January 2021. The inclusion period extended more than expected because only women at <20 weeks of gestational age were included. There were only few such cases in the study health centers because women usually start their antenatal visits late in Burkina Faso. As women were to be followed up until 6 weeks postpartum, the last data collection interview ended in September 2022. Data analysis was completed in June 2023, and the results are expected to be published in February 2024.

## Discussion

### Study Overview

With the rise of information and communication technologies and the WHO’s recommendations for the use of mobile technology in antenatal health care, several clinical trials using mobile health apps have been conducted with varied results. Most of these clinical trials [12-17] used a mobile text messaging system for sending awareness and information messages to pregnant women. Those studies proposed to measure pregnant women’s knowledge and the quality of ANC. Apart from mobile apps, some computerized clinical decision support systems have been implemented. Haddad et al [35] conducted a systematic review of these computerized systems in 2019 and found 9 systems that met their eligibility criteria, including the PANDA system. The PANDA system was found to be one of the most comprehensive apps with several features. However, those authors concluded that the use of these systems for ANC was limited [35]. Adepoju et al [36] conducted a systematic review of clinical decision support systems in sub-Saharan Africa in 2017 and reached the same conclusion. However, they noted that clinical decision support systems could improve patient-provider relationships by building trust in the provision of health services. They also noted that although health care workers are generally enthusiastic about using these types of systems, there are concerns about the effects of increased workload, workflow changes, and technical challenges. These different barriers to implementing health apps were also found by Zakane et al [37] in Burkina Faso. Thus, the added value of these systems remains to be proven. Although studies on the PANDA system have shown that it can provide standardized ANC as per the WHO’s guidelines [14], it is still essential to evaluate its intrinsic capacity to improve the quality of care by conducting a high-level evidence study. In this sense, the randomized trial that will be conducted in our research will be of crucial importance because it will help to demonstrate whether, in addition to offering standardized care, the PANDA system allows for an improvement in the quality of care provided to pregnant women, their satisfaction, and their knowledge on birth preparedness and complication readiness. Moreover, considering that Burkina Faso is a country with limited resources, such health policies could be cost-effective; hence, the economic evaluation carried out throughout the implementation is pertinent and essential. In addition, the results of this trial will be significant because computerized clinical decision support systems are anticipated to be implemented in Burkina Faso in the future in several fields, including reproductive health (through the electronic consultation register for maternal care) and child health (through the electronic consultation register for the management of child diseases).

### Strengths and Limitations of This Study

The limitations and challenges of this complex intervention include difficulties in coordinating with study sites and challenges related to the COVID-19 pandemic in implementing the intervention, maintaining its fidelity, and keeping the trial on schedule. Although we have designed a cluster randomized controlled trial, neither the study sites nor the participants are blinded to the study conditions because of the nature of the intervention. Moreover, the participating sites are limited to 1 rural district of Burkina Faso; thus, the results may not be generalizable at the national level (no urban facilities are included in this study). However, our results could guide countrywide policies for improving maternal and newborn health, highlighting the benefits of such an electronic decision support system for similar rural African areas where maternal and newborn morbidity and mortality rates are high.

### Conclusion

This study will help to determine the proportion of women who receive quality ANC as recommended in clinical standards and protocols and whether electronic registries can help improve ANC in a context of low-quality human resources, like in Burkina Faso. This study’s qualitative research and economic evaluation will identify the barriers and facilitators of such an app in resource-limited countries such as Burkina Faso and contribute to the cost-effectiveness analysis of 2 strategies (PANDA vs usual care). In a randomized study design, the results will likely provide a high level of evidence compared to the results already available in the literature, which are from pilot or feasibility studies with smaller sample sizes.

## Appendix

Multimedia Appendix 1 Data collection instrument.

## Abbreviations

ANC: antenatal care
EDSBF-MICS: Burkina Faso Enquête Démographique et de Santé et à Indicateurs Multiples
ICC: intraclass correlation coefficient
PANDA: Pregnancy and Newborn Diagnostic Assessment
WHO: World Health Organization

## Data Availability

The data sets generated and analyzed during this study will be available from the corresponding author upon reasonable request following the completion of the trial and publication of the primary outcome paper.
