# Supplementary material for: Effects of the Pregnancy and Newborn Diagnostic Assessment (PANDA) App on Antenatal Care Quality in Burkina Faso: Protocol for a Cluster Randomized Controlled Trial
Source: JMIR Res Protoc. 2023 Aug 9;12:e37136. doi: 10.2196/37136 (PMC10448280; doi:10.2196/37136)
Supplement: Multimedia Appendix 1 [file resprot_v12i1e37136_app1.pdf]

Data collection instrument
Screening form:
Q1. Date of screening
INCLUSION CRITERIA: Questions completed by the PROVIDER
Q2 - Q3: are
Q4, Q5, Q6 are
Q2. (Estimated by the Provider) Is the estimated age of pregnancy less than 16 weeks of amenorrhea? Yes ☐ No ☐
Q3. (Estimated by the Provider) Does the health status of the woman and her pregnancy allow for delivery at the health centre (no need to refer to higher level, etc) Yes ☐ No ☐
Q4. (Answered by the woman) Are you willing to follow up your pregnancy at this health centre? Yes ☐ No ☐
Q5. (Answered by the woman) Do you intend to give birth in this health centre? Yes ☐ No ☐
Q6. (Answered by the woman) Is this the only study you will participate in? Yes ☐ No ☐

If all the answers of Q2 - Q6 are YES, then refer the woman to the data collector for the informed consent process after offering antenatal care.

INCLUSION CRITERIA: Question completed by the data collector
Q7. After giving all relevant information about this study and the follow-up to the woman, does the woman give informed consent to participate in the study? Yes ☐ No ☐ If Yes, then the woman is eligible.
Q8. (Question completed by data collector) Is the woman eligible to participate in the study? Yes ☐ No ☐
If the answer to Q8 = Not eligible, reassure the woman that she will not be discriminated against during her care at the health centre, then end the questionnaire and offer her antenatal care.
If the answer to Q8 = Eligible, the interviewer continues the interview by completing the GINFOS forms.

PARTICIPANT'S GENERAL INFORMATION FORM: (completed the day of the inclusion of the woman)
Q1. Date of interview
Q2. How old are you? 99 if don't know
Q3. Where do you live?
1. Rural or urban-rural
2. Urban

Q4. Name of village (or sector if urban) ....
Q5. What is the highest level of education you have achieved?
1. Never attended
2. Primary

3. Secondary
4. Higher

Q5. What is your current occupation?

1. None
2. Pupil / Student
3. Housewife / farmer
4. Employee
5. Trader
6. Craftswoman
7. Other

Q6. If other occupation, specify ....

Q7. What is your husband's approximate age in years?

Q8. What is the highest level of education achieved by your husband?

1. Never attended
2. Primary
3. Secondary
4. Higher

Q9. What is your husband's occupation, i.e. what type of work does he do mainly to earn an income?

1. None
2. Pupil/student
3. Housewife / farmer
4. Employee
5. Trader
6. Craftswoman
7. Other

Q10. If other occupation, specify ....

Q11. Do you live in a joint family or with an in-law?

1. No, only in a nuclear family
2. Yes, with members of the in-laws

Q12. How long does it usually take you to get to the health centre from your home?

Q13. How far is your home from the health centre? |____| km |_______| metres

Q14. What is your current marital status?

1. Married or in union
2. Single

Q15. Do you leave your home alone whenever you need to consult or go to a health centre for any reason?

1. Yes, I always go alone
2. No, I am often accompanied
3. No, I am always accompanied

Q16. If the answer is often (or always) accompanied, who usually accompanies you when you go to a health centre?

1. Mother-in-law
2. Father-in-law
3. Sister-in-law or brother-in-law
4. Mother or sister

5. Other (specify)

Q17. If other, specify ....

Q18. How many pregnancies in total have you had, including the current one?

Q19. How many children were born alive?

Q20. How many stillbirths have you had?

Q21. How many abortions have you had?

Q22. How many living (biological) children do you have?

Q23. How old is your last living child? |____| year |_______| month

Q24. Have you ever breastfed?

0. No
1. Yes

Q25. How long did you exclusively breastfeed your last child? (without giving any other supplements) |____| month |_______| day

Q35. Approximately how long was it between the end of your last pregnancy and the beginning of your current pregnancy? |____| year |_______| month

Q26. Was this current pregnancy planned?

0. No
1. Yes
2. Don't know

Q27. What was the outcome of your last pregnancy?

1. Abortion
2. Stillbirth
3. Live birth
4. One born alive and one deceased (if twin)

Q28. Were you using a family planning method in the year before you became pregnant with this current pregnancy?

0. No
1. Yes

Q29. If no, why? List some reasons
(record 1 if reason given and 0 if not)

A. Sex infrequent/no sex
B. Spouse on the move
C. I want as many children as possible
D. I am opposed to FP
E. Spouse opposed
F. Other people opposed
G. Religious opposition
H. I do not know any method
I. I have health problems
J. I am afraid of the side effects
K. Geographical inaccessibility/too far away
L. Financial inaccessibility/expensive methods
M. Don't know
N. Other reason

Q30. If other reason, specify ....

Q31. If yes to question 38, what FP method did you use?

1. LAM

2. Injectable
3. Pill
4. IUD (or intrauterine device)
5. Male condom
6. Female condom
7. Implant
8. Emergency contraception
9. Vaginal spermicide
10. Female sterilisation
11. Vasectomy
12. Withdrawal
13. Abstinence
14. Fixed days (cycle necklace, calendar)
15. No method
16. Other method

ASSESSING WOMEN'S AUTONOMY IN DECISION-MAKING

Now I am going to ask you about general decision-making in your household. For each of the following situations, tell me who makes the final decision: yourself, your husband, mother-in-law, father-in-law, or any other person. You can also name more than one person if the decision is made collectively.

Q32. For all the items below, mentions who decides, between: Yourself, your spouse, your Mother-in-law, your Father-in-law, or Other person

A. small household expenses such as toothpaste, soap, dishes, etc.
B. major household expenses such as television, radio, refrigerator, etc.
C. women's expenses such as clothes and jewellery
D. your job outside the home (the job you want)
E. buying children's clothes
F. where to go for advice if the child is ill
G. where to go to see a doctor if you are ill
H. buying medicines
I. visiting your parents (when or where)
J. the number of children in your couple
K. using the family planning method

ASSESSMENT OF SOCIO-ECONOMIC LEVEL

Read to the respondent: "I would like to ask you some questions about your living conditions. I realise that some of these questions do not seem to be related to health care, but they all help us to understand your living situation. Please answer as honestly as possible.

Read the questions to the respondent exactly as they are written. Do not read the answer options. All questions must be answered.

Q33. How many people in total are in the household?

Q34. How many members of the household are under 5 years of age?

Q35. How many household members are between 5 and 16 years old (including 16 years old)?
Q36. How many children between 5 and 16 years old go to school?
Q37. Does the household have a refrigerator or freezer?
0. No
1. Yes
Q38. Does the household have a television?
0. No
1. Yes
Q39. Does the household have electricity?
0. No
1. Yes
Q40. Does the household have a radio?
0. No
1. Yes
Q41. Does the household have a telephone?
0. No
1. Yes
Q42. Number of motorbikes in the household
Q43. Number of tricycles in the household
Q44. Number of household cars
Q45. Where does the water that your household members drink come mainly from?
1. tap water
2. Open well
3. Covered well or borehole
4. Surface water (dam, river/river, pond/lake)
5. Rainwater
6. Tanker truck
Q46. If other, specify ....
Q47. What type of toilet do most members of your household use?
1. Flush toilet
2. Rudimentary pit/latrine
3. Improved pit/ latrine
4. No toilet/nature
5. Other
Q48. If other, specify ....
Q49. Do you share this facility with other households?
0. No
1. Yes
Q50. In your household, what kind of fuel do you mainly use for cooking?
1. electricity
2. Bottled gas/natural gas
3. Charcoal
4. Firewood, straw
5. Dung
6. Other
Q51. If other fuel, specify ....

Q52. What is the main material of the walls

1. Banco
2. Wood/board
3. Tent
4. Cement or stone
5. Other

Q53. If other wall material, specify ....

Q54. What is the main material of the floor

1. Earth/sand
2. Wood/planks
3. Cement
4. Tiles
5. Other

Q55. If other floor material, specify ....

CHILDBIRTH AND EMERGENCY PREPAREDNESS PLAN KNOWLEDGE FORM: CPAU1

Q1. How many prenatal visits should a pregnant woman make in total?

Q2. Give the recommended frequency of ANCs

Q3. Are you familiar with the PAU card?

0. No
1. Yes

Q4. If yes, have you ever received explanations about the images on this map?

0. No
1. Yes

Q5. Do you own this card?

0. No
1. Yes

Q6. If yes, how many?

Q7. Has it been discussed with your spouse?

0. No
1. Yes

Q8. Have you identified a care facility for your delivery or in case of emergency?

0. No
1. Yes

Q9. Have you identified an attendant for the birth or in case of emergency?

0. No
1. Yes

Q10. If yes, what is the relationship to you?

1. my spouse
2. My mother
3. My mother-in-law
4. A sister/brother
5. A sister-in-law/brother-in-law
6. My co-wife
7. A friend
8. Other person

Q11. If other person, specify ....
Q12. How do you plan to finance expenses related to your delivery or in case of emergency?

1. I haven't thought about that yet
2. Don't know
3. My husband will take care of it
4. Personal savings (bank, tontine, etc)
5. I plan to sell some property
6. Health insurance
7. Health insurance or cost-sharing scheme
8. Family solidarity
9. Other

Q13. If other planning, specify ....
Q14. What means of transport have you identified to get to the health facility during delivery or in case of an emergency?

1. On foot
2. By bicycle
3. By motorbike
4. By car
5. I haven't thought about it yet
6. Don't know
7. My husband is in charge of it
8. Other

Q15. If other, specify ....
Q16. What materials did you remember to bring in case of delivery?

1. clean loincloths
2. Linen for the baby
3. Drinks
4. Toiletries
5. Delivery kit
6. Clean linens
7. Placenta collection jar
8. Other

Q17. If other equipment, specify ....
Q18. Have you identified a blood donor for a possible need during delivery or in case of emergency?

0. No
1. Yes

Q19. If yes, how are you related to the potential blood donor?

1. my spouse
2. My mother
3. My mother-in-law
4. A sister/brother
5. A sister-in-law/brother-in-law
6. My co-wife
7. A friend
8. Other person

Q20. If other person, specify ....

Q21. Do you know of any danger signs of pregnancy?

0. No
1. Yes

Q22. If yes, which ones?

1. Vaginal bleeding
2. Fever
3. Intensive unresponsive headache
4. Difficulty breathing
5. Convulsions and/or loss of consciousness
6. Severe abdominal pain
7. Non-bloody vaginal discharge

Q23. What care does a newborn need?

1. keep the newborn away from mosquitoes
2. Keep the newborn away from animals
3. Controlling the chill of the newborn (skin-to-skin contact, kangaroo method, etc)
4. Early breastfeeding of the newborn (within 30 minutes of birth)
5. Other

Q24. If other care, specify ....

Q25. Do you know of any danger signs of pregnancy?

0. No
1. Yes

Q26. If yes, which ones?

1. Vaginal bleeding
2. Fever
3. Intensive unresponsive headache
4. Difficulty breathing
5. Convulsions and/or loss of consciousness
6. Severe abdominal pain
7. Non-bloody vaginal discharge

Q27. Do you know the signs of early labour?

0. No
1. Yes

Q28. Name the signs of the onset of labour

1. Water breaking
2. Regular painful contractions
3. Loss of red, viscous mucus

Q29. Do you know the danger signs for newborns?

0. No
1. Yes

Q30. Name some danger signs in newborns

1. Fever
2. Hypothermia
3. Convulsions
4. Inability to suckle
5. Vomiting

6. Pus at the cord
7. Purulent discharge from the eyes
8. Icterus
9. Hypotonia
10. Unexplained crying

Q31. Does your husband accompany you to the CSPS when you go for a consultation?

1. Never
2. Yes, rarely
3. Yes, often
4. Yes, always

First antenatal care quality form (completed the day of the inclusion of the woman)

For all questions in this section, write 1 if the action was performed, if the question was asked, if the advice was given, or if the treatment was performed (this form is administered each time the participant comes to the health centre for antenatal care).

Q1. Date of visit
Q2. Type of visit 1st PNC
Q3. Permission for spouse to attend consultation
Q4. If yes, spouse attended the consultation
Q5. Possession of a health booklet
Q6. Alcohol consumption requested
Q7. Smoking requested
Q8. Ultrasound prescribed and or performed (Consult the woman's health record for this question)
Q9. Urine sugar and albumin test (Refer to the woman's health record for this question)
Q10. Blood count or BH test (Consult the woman's health record for this question)
Q11. Hepatitis B vaccination checked and/or performed
Q12. Blood glucose test performed (Consult the woman's health record for this question)
Q13. Blood pressure taken (Consult the woman's health record for this question)
Q14. Search for oedema of the feet and face (Consult the woman's health record for this question)
Q15. Weight taken (Consult the woman's health record for this question)
Q16. Temperature taken (Consult the woman's health record for this question)
Q17 Physical examination: Uterine height measured (Consult the woman's health record for this question)
Q18. Physical examination: Vaginal touch performed (Consult the woman's health record for this question)
Q19. Physical examination: Decision on obstetrical prognosis (normal pelvis, borderline pelvis, BGR, etc) made (Consult the woman's health record for this question)
Q20. Tetanus vaccination checked and/or performed (Consult the woman's health record for this question)
Q21. Malaria prophylaxis (sulfadoxine-pyrimethamine) prescribed

Q22. Malaria prophylaxis: woman correctly explains dosage
Q23. Anti-anemia prophylaxis (iron-folic acid) prescribed
Q24. Antimalarial prophylaxis: woman explains dosage correctly
Q25. Antiparasitic prophylaxis prescribed
Q26. Parasite prophylaxis: woman explains dosage correctly
Q27. Danger signs of pregnancy explained to woman
Q28. The place of delivery was discussed
Q29. Importance of exclusive breastfeeding was given
Q30. Early initiation of breastfeeding was advised
Q31. Mother's feeding: good feeding practices discussed
Q32. Contraception in the postpartum period was discussed
Q33. The use of an impregnated mosquito net was explained
Q34. Newborn care explained
Q35. Birth preparation plan discussed
Q36. The woman was given the opportunity to ask questions if necessary
Q37. An appointment was negotiated
Q38. Summary of the antenatal care results written in woman's health record
Refer to the woman's health record for this question

Subsequent antenatal care quality form (completed at every subsequent antenatal visit of the woman)

For all questions in this section, write 1 if the action was performed, if the question was asked, if the advice was given, or if the treatment was performed (this form is administered each time the participant comes to the health centre for antenatal care) and 0 if not.

Q1. Date of visit
Q2. Type of visit
Q3. Partner's presence at the visit
Q4. Prenatal consultation
Q5. Possession of a health record
Q6. Alcohol consumption requested
Q7. Smoking requested
Q8. Ultrasound performed
Q9. Urine sugar and albumin test
Q10. Blood count or BH test
Q11. Blood glucose test performed
Q12. Urine cytobacteriological examination performed
Q13. Blood pressure taken
Q14. Fetal heart sounds recorded
Q15. Search for oedema of the feet and face
Q16. Weight measurement
Q17. Temperature taken
Q18. Physical examination: Uterine height measured
Q19. Physical examination: Vaginal touch performed

Q20. Physical examination: Obstetrical prognosis decision (normal pelvis, borderline pelvis, BGR, etc) made
Q21. Tetanus vaccination checked and/or performed
Q22. Hepatitis B vaccination checked and/or performed
Q23. Anti-anaemic prophylaxis (iron-folic acid) prescribed
Q24. Antemalarial prophylaxis: woman explains dosage correctly
Q25. Anti-malarial prophylaxis prescribed
Q26. Anti-malarial prophylaxis: woman explains dosage correctly
Q27. Danger signs of pregnancy explained to woman
Q28. Place of delivery discussed
Q29. The importance of exclusive breastfeeding was given
Q30. The importance of exclusive breastfeeding was discussed
Q31. The mother's diet: good feeding practices were discussed
Q32. Contraception in the postpartum period was discussed
Q33. The use of an impregnated mosquito net was explained
Q34. Newborn care was explained
Q35. The birth preparation plan was discussed
Q36. The woman was asked to raise concerns if necessary
Q37. An appointment was negotiated
Q38. Summary of the antenatal care results in the woman's health card

SATISFACTION FORM : (completed the day of the inclusion of the woman and at every subsequent antenatal visit)
Likert scale, 0 to 4, 0=no satisfaction, 4= strongly satisfied)
INTERPERSONAL RELATIONS WITH STAFF (5 sub-items)
Q1. Reception/Greetings of providers
Q2. Respecting the confidentiality of the consultation with the provider
Q3. Respect for privacy during consultation
Q4. Respect and courtesy of providers during NPS
Q5. Goodbye from staff
QUALITY OF SERVICE (9 sub-items)
Q6. Waiting time
Q7. Overall time spent in the health centre
Q8. Other times to wait for test results
Q9. Skills
Q10. Explanation of iron+folic acid dosage
Q11. Explanation of malaria prophylaxis
Q12. Explanation of preventive measures against malaria
Q13. Explanation of the woman's diet (foods rich in iron and folic acid, vitamin C and proteins)
Q14. Explanation of the different complementary examinations

DELIVERY FORM: (**completed at childbirth’s day)**
Q1. Date of delivery

Q2. Place of delivery

1. Study health centre
2. Other health facility
3. Home and in the presence of service providers
4. Home and no service providers
5. Other

Q3. If other place of delivery, specify ....

Q4. Main reason why the participant did not deliver at the study health facility

1. Referred to another health facility
2. Geographic accessibility
3. Cost of delivery
4. Lack of transport
5. Traditional customs and practices
6. Poor quality of care at the health centre
7. Refusal of partner/family
8. Other

Q5. If other, specify ....

Q6. Mode of delivery:

1. Vaginal delivery, normal (not instrumented)
2. Low level, instrumented (e.g. forceps, vacuum)
3. Caesarean section

Outcome of delivery

1. Alive and well
2. Living with a health problem
3. Stillborn

Q7. Describe the health problem of the newborn

Q8. Status of the participant after delivery

1. Alive
2. Maternal death

Q9. Presence of husband at CSPS during delivery

0. No
1. Yes

Q10. Presence of the husband in the delivery room during delivery

0. No
1. Yes

Q11. Do you use a family planning method?

0. No
1. Yes

Q12. If yes, what FP method do you use?

1. LAM
2. Injectable
3. Pill
4. IUD (or intrauterine device)
5. Male condom
6. Female condom
7. Implant
8. Emergency contraception

9. Vaginal spermicide
10. Female sterilisation
11. Vasectomy
12. Withdrawal
13. Abstinence
14. Fixed days (cycle necklace, calendar)
15. No method
16. Other method

Q13. If other method, specify ....

Q14. Did you decide to use a family planning method during the first year after giving birth?

0. No
1. Yes
2. Don't know

Q15. How soon after delivery do you plan to start your chosen method?

1. 0 - 14 days after delivery
2. 2 - 8 weeks after delivery
3. 3 - 6 months after delivery
4. More than 6 months after the birth
5. Don't know

Q16. If yes, what is the main method you can think of?

1. LAM
2. Injectable
3. Pill
4. IUD (or intrauterine device)
5. Male condom
6. Female condom
7. Implant
8. Emergency contraception
9. Vaginal spermicide
10. Female sterilisation
11. Vasectomy
12. Withdrawal
13. Abstinence
14. Fixed days (cycle necklace, calendar)
15. No method
16. Other method

Exit form (completed at the end of the follow-up, normally at six weeks postpartum, or if the woman is lost to follow-up, or refused the continue the study or have an abortion)

Q1. Date of completion of the study form

Q2. Date of completion of study (see instructions)

Q3. Status of the participant

1. study completed

2. Refused to continue the study (End the questionnaire)
3. Lost to follow-up (End questionnaire)
4. Death of the woman (End questionnaire)
5. Abortion (End questionnaire)
6. Miscarriage (End questionnaire)

Q4. If "Refused to continue study", what was the main reason?
1. Personal reason
2. Health problem
3. No motivation
4. Other

Q5. if other, specify ---

Q6. Status of the child
1. Living
2. Deceased
3. One living, one dead
4. Stillborn
5. Abortion
6. Other

Q7. If other, specify --

Q8. Do you use a family planning method?
0. No
1. Yes

Q9. If yes what FP method do you use?
1. LAM
2. Injectable
3. Pill
4. IUD (or intrauterine device)
5. Male condom
6. Female condom
7. Implant
8. Emergency contraception
9. Vaginal spermicide
10. Female sterilisation
11. Vasectomy
12. Withdrawal
13. Abstinence
14. Fixed days (cycle necklace, calendar)
15. No method
16. Other method

Q10. If other method, specify ....

Q11. Did you decide to use a family planning method during the first year after giving birth?
0. No
1. Yes
2. Don't know

Q12. How soon after the birth do you plan to start your chosen method?
1. 0 - 14 days after the birth

2. 2 - 8 weeks after delivery
3. 3 - 6 months after delivery
4. More than 6 months after the birth
5. Don't know

Q13. If yes, what is the main method you are thinking of?

1. LAM
2. Injectable
3. Pill
4. IUD (or intrauterine device)
5. Male condom
6. Female condom
7. Implant
8. Emergency contraception
9. Vaginal spermicide
10. Female sterilisation
11. Vasectomy
12. Withdrawal
13. Abstinence
14. Fixed days (cycle necklace, calendar)
15. No method
16. Other method

Q14. If other, specify ---
